# Ac-SDKP attenuates ER stress-stimulated collagen production in cardiac fibroblasts by inhibiting CHOP-mediated NF-κB expression

**DOI:** 10.3389/fphar.2024.1352222

**Published:** 2024-03-01

**Authors:** Hamid Suhail, Hongmei Peng, Khalid Matrougui, Nour-Eddine Rhaleb

**Affiliations:** ^1^ Department of Internal Medicine, Hypertension and Vascular Research Division, Henry Ford Hospital, Detroit, MI, United States; ^2^ Department of Physiology Sciences, Eastern Virginia Medical School, Norfolk, VA, United States; ^3^ Department of Physiology, Wayne State University, Detroit, MI, United States

**Keywords:** Ac-SDKP, ER stress, UPRs, inflammation, collagen, human cardiac fibroblast

## Abstract

Inflammation and cardiac fibrosis are prevalent pathophysiologic conditions associated with hypertension, cardiac remodeling, and heart failure. Endoplasmic reticulum (ER) stress triggers the cells to activate unfolded protein responses (UPRs) and upregulate the ER stress chaperon, enzymes, and downstream transcription factors to restore normal ER function. The mechanisms that link ER stress-induced UPRs upregulation and NF-κB activation that results in cardiac inflammation and collagen production remain elusive. N-Acetyl-Ser-Asp-Lys-Pro (Ac-SDKP), a natural tetrapeptide that negatively regulates inflammation and fibrosis, has been reported. Whether it can inhibit ER stress-induced collagen production in cardiac fibroblasts remains unclear. Thus, we hypothesized that Ac-SDKP attenuates ER stress-stimulated collagen production in cardiac fibroblasts by inhibiting CHOP-mediated NF-κB expression. We aimed to study whether Ac-SDKP inhibits tunicamycin (TM)-induced ER stress signaling, NF-κB signaling, the release of inflammatory cytokine interleukin-6, and collagen production in human cardiac fibroblasts (HCFs). HCFs were pre-treated with Ac-SDKP (10 nM) and then stimulated with TM (0.25 μg/mL). We found that Ac-SDKP inhibits TM-induced collagen production by attenuating ER stress-induced UPRs upregulation and CHOP/NF-κB transcriptional signaling pathways. CHOP deletion by specific shRNA maintains the inhibitory effect of Ac-SDKP on NF-κB and type-1 collagen (Col-1) expression at both protein and mRNA levels. Attenuating ER stress-induced UPR sensor signaling by Ac-SDKP seems a promising therapeutic strategy to combat detrimental cardiac inflammation and fibrosis.

## Introduction

Cardiac inflammation and fibrosis are significant factors in hypertension and cardiac remodeling, leading to heart failure. The endoplasmic reticulum (ER) is the primary cell organelle involved in translating, assembling, and folding at least 35% of new secretory proteins (Pejman et al., 2020; Stauffer et al., 2020). Physiologically, ER is an early target of stress (Zhao and Ackerman, 2006; Ron and Walter, 2007). Any change that disturbs ER homeostasis results in the accumulation of unfolded/misfolded proteins in the ER lumen (Oslowski and Urano, 2011; Galan et al., 2014), which causes an overload burden, a condition known as ER stress (Galan et al., 2014). Current evidence indicates that prolonged ER stress and UPR activation initiate inflammation and fibrotic remodeling in several end organs, including the heart, kidney, liver, and lung (Tanjore et al., 2013; Burman et al., 2018; Kropski and Blackwell, 2018; Amen et al., 2019). ER chaperone regulates the synthesis of Col-1. The impaired ER chaperon causes high levels of type −1 collagen (Col-1), the main fibrillary collagen content found in cardiac fibrosis, ventricular stiffness, hampered contraction and relaxation, and cardiac dysfunction (Ayala et al., 2012). Global studies have also demonstrated a positive correlation between infiltrating pro-inflammatory macrophages and myocardial fibrosis in cardiovascular diseases, hypertension, and heart failure (Boluyt et al., 1994). IL-6 is one of the critical mediators that induces inflammation, and we previously reported that IL-6 gene deletion prevents cardiac inflammation, fibrosis, and dysfunction (Gonzalez et al., 2015). The detrimental role of ER stress is found in many pathophysiological conditions, including hypertension and other cardiovascular diseases (Peng et al., 2010). Unraveling ER stress mechanisms is essential to identify and understand potential therapeutic targets in cardiovascular remodeling and dysfunction. Globally, *in vitro* and *in vivo* studies have highlighted the role of ER stress in hypertension and cardiac fibrosis leading to heart failure; however, the mechanism remains elusive. The overload proteins burden in ER-lumen triggers the activation of unfolded protein response (UPR), an adaptive mechanism generated by the cells that activates three UPR-sensitive signaling pathways, namely 1) inositol-requiring enzyme type 1 (IER1), 2) protein kinase RNA-like endoplasmic reticulum kinase (PERK) and 3) activating transcription factor 6 (ATF6). Pharmacological compounds such as tauroursodeoxycholic acid (TUDCA) and 4-phenyl butyric acid are prevalent drugs used to counteract ER stress in both *in vivo* and *in vitro* experimental models (Galan et al., 2014). N-Acetyl-Ser-Asp-Lys-Pro (Ac-SDKP) is an endogenous tetrapeptide released from its precursor thymosin-*β*_4_ by two successive enzymes meprin-α and prolyl oligopeptidase (Cavasin et al., 2004; Peng et al., 2014; Kumar et al., 2016; Kumar and Yin, 2018; Kassem et al., 2019). Ac-SDKP was initially isolated from fetal calf bone marrow by Lenfant *et al.* and reported as a ubiquitous endogenous peptide (Lenfant et al., 1989). However, later studies have shown that Ac-SDKP is widely distributed in other tissues, including bone marrow, lungs, kidneys, hearts, and circulating mononuclear cells (Pradelles et al., 1991; Rhaleb et al., 2001; Liu et al., 2009). Ac-SDKP is mainly found in tissues with angiotensin converting enzyme (ACE). Ac-SDKP is hydrolyzed exclusively by the ACE N-domain rather than the ACE C-domain (Rousseau et al., 1995; Azizi et al., 1996; Rasoul et al., 2004). The half-life of Ac-SDKP is 4.5 min in human plasma and, thus, is probably released continuously (Azizi et al., 1997; Rasoul et al., 2004). Ac-SDKP was originally described as a natural suppressor of pluripotent hematopoietic stem cell proliferation (Lenfant et al., 1989; Azizi et al., 1996; Sharma et al., 2008). Still, recent studies have shown a relatively broad inhibitory spectrum of the tetrapeptide, including cardiac, renal, and lung fibroblasts, mesangial cells, vascular smooth muscle cells, and hepatocytes (Lombard et al., 1990; Iwamoto et al., 2000; Pokharel et al., 2002; Kanasaki et al., 2003; Sun et al., 2010; Xu et al., 2012). We have previously shown that the ACE inhibitor (ACEi), captopril, significantly prevented Ac-SDKP degradation and increased its plasma level fivefold in volunteers, inhibiting the hydrolysis of ^3^H-Ac-SDKP in circulation by 90%–99% (Azizi et al., 1996; Azizi et al., 1997; Rhaleb et al., 2001). Despite years of research and many studies demonstrating the pharmacological and physiological effects of Ac-SDKP, the mechanisms that mediate those effects are not fully understood. We previously reported that Ac-SDKP suppresses TNF-α-induced ICAM expression by inhibiting IκB kinase and NF-κB activation (Zhu et al., 2016). However, whether Ac-SDKP interferes with the mechanisms through which ER stress-activated CHOP/NF-κB signaling contributes to inflammation and the enhanced collagen production in cardiac fibroblast is unknown. Therefore, we hypothesized that Ac-SDKP attenuates ER stress-stimulated collagen production in cardiac fibroblasts by inhibiting CHOP-mediated NF-κB expression. Here, we tested whether Ac-SDKP inhibits tunicamycin (TM, a known potent ER stress inducer)-induced ER stress signaling, NF-κB signaling pathway, release of inflammatory cytokine IL-6, and collagen production in human cardiac fibroblasts (HCFs).

## Materials and methods

### Reagents and antibodies

Human cardiac fibroblasts (HCFs), fibroblast basal media (FBM), and the respective growth factors were purchased from Lonza (Walkersville, MD, USA). Ac-SDKP was purchased from Genescript Biotech Corp (Piscataway, NJ, USA), and purity was 98.4% (GenScript, Piscataway, NJ). TUDCA, TM, and captopril (an ACE inhibitor) were purchased from Sigma-Aldrich (St. Louis, MO, USA). Protease and phosphatase inhibitors for protein preparation were purchased from Roche (Roche Diagnostics, Indianapolis, IN, USA). The antibodies against IRE1α (cat#3294), XBP-1 (cat#40435), ATF4 (cat#11815), CHOP (cat#2895), NF-κB (cat#8242), and GAPDH (cat#2118) were purchased from Cell Signaling Technology (Danvers, MA, USA). Type-1 collagen antibody (Cat#600-401-103) was purchased from Rockland Immunochemicals, Inc. (Limerick, PA, USA). The primers used for RT-PCR analysis were purchased from Eurofins Genomics LLC (Louisville, KY, USA) and their corresponding sequences are listed in Table 1. CHOP-specific shRNA in pGFP-C-shLenti vector as Lentiviral plasmids format was purchased from OriGene (Rockville, MD, USA).

**TABLE 1:** List of the primers used to quantify RT-PCR.

S. No	Gene	Forward sequence (5^′^-----3^′^)	Reverse sequence (5^′^-----3^′^)
1	IRE1α	TAG​TTT​GTT​GCC​TCT​GGG​ATT​AG	GGA​TCA​GCG​TTA​TCC​TCT​TCT​G
2	XBP-1	ACCCCGCCCGAGTTGA	GCG​GGT​ATA​TTC​ATC​ACT​TAT​TGG​T
3	ATF4	ATG​GCC​GGC​TAT​GGA​TGA​TG	TCT​GGC​ATG​GTT​TCC​AGG​TC
4	CHOP	GGA​ACC​TGA​GGA​GAG​AGT​GTT​C	AAG​GTG​AAA​GGC​AGG​GAC​TC
5	NF-κB	CGC​AAA​AGG​ACC​TAC​GAG​AC	TGG​GGG​AAA​ACT​CAT​CAA​AG
6	IL-6	TTC​CAT​CCA​GTT​GCC​TTC​TTG	TTG​GGA​GTG​GTA​TCC​TCT​GTG​A
7	Col-1	CAT​GTT​CAG​CTT​TGT​GGA​CCT	GCA​GCT​GAC​TTC​AGG​GAT​GT
8	L27	ACA​TTG​ACG​ATG​GCA​CCT​C	GCT​TGG​CGA​TCT​TCT​TCT​TG

### Cell culture and treatments

HCFs from passages 3-6 were maintained at 37°C, 5% CO_2,_ and 95% air in low glucose fibroblast basal media supplemented with 10% fetal bovine serum (FBS) and fibroblast growth factors. The media was changed every 2–3 days until cells reached 80%–90% confluence. Before the experiments, the cells were starved overnight in the serum-free basal medium at 37°C. Before the treatments, 1 µM captopril was added to the medium of all groups, including the control, to prevent the breakdown of Ac-SDKP by ACE (Grillon et al., 1993). We previously reported that Ac-SDKP at 10 nM is an appropriate dose for our pharmacological studies (Rhaleb et al., 2001; Zhuo et al., 2007). Thus, the cells were treated in 0.5% FBS containing basal media with PBS (control), TM (0.25 μg/mL), Ac-SDKP (10 nM) plus TM, and TUDCA (500 μg/mL) plus TM. Cells were pretreated with Ac-SDKP and TUDCA for 30 min before adding TM.

### Cell viability and proliferation assay

Cell proliferation was determined using a water-soluble tetrazolium salt (WST-1) kit as instructed by the manufacturer (Quick Cell proliferation, Bio Vision Waltham, MA). Briefly, HCFs were seeded in a 96-well microtiter plate in a final 100 µL/well volume at a 15–20 × 10^3^ cells/well density. After 24 h of treatment, 10 µL of WST-1 solution was added to each well for 4 h at standard cell culture conditions. The formazan dye produced from active live cells was quantified at 440 nm by a spectrophotometer.

### Lentiviral shRNA interference

These ready-to-transduce CHOP-specific Lentiviral shRNA vectors include an shRNA expression cassette driven by a U6 promotor, the puromycin resistance marker driven by an SV40 promotor, and tGFP driven by a CMV promotor. 0.3 × 10^6^ cells were seeded in a six-well plate and grown for 24 h at 37°C, 5% CO_2,_ and a 95% air incubator. The cells at 50%–60% confluence were transfected with shRNA CHOP and incubated for 48 h for RNA analysis or 72 h for protein analysis according to ORIGENE recommendation. After transfection, the media was replaced with complete basal media, and the cells were grown for 24 h at 37°C, 5% CO_2,_ 95% air incubator. After 24 h, the cells were pretreated with Ac-SDKP (10 nM) and TUDCA (500 μg/mL) for 30 min and then stimulated with TM (0.25 μg/mL).

### Western blot analysis

The cell lysates were prepared using a Mammalian Cell Lysis Kit (cat#MCL1, Sigma-Aldrich) with an added “PhosSTOP” (ref#04906845001, Roche Diagnostic GmbH, Mannheim, Germany) and protease inhibitor cocktail (cat# 200-664-3, Sigma-Aldrich) as we previously reported (Liu et al., 2009). The Bradford Protein Assay (Bio-Rad) was performed to measure the protein concentration. Briefly, proteins (30 µg) were subjected to electrophoresis on 4%–15% SDS-PAGE under reducing conditions and electro-transferred to nitrocellulose membranes (Bio-Rad Laboratories). The membranes were blocked in 5% fat-free milk for 2 h and probed with primary antibodies against IRE1α, XBP-1, CHOP, ATF4, NF-κB, Col-1, and GAPDH with a dilution of 1:2,500 overnight at 4°C on a shaking platform. Horseradish peroxidase (HRP)-conjugated anti-rabbit IgGs or anti-mouse IgGs (Cell Signaling Technology) was used to visualize proteins by a chemiluminescence reaction (Bio-Rad Laboratories, Hercules, CA). The protein expression was normalized to GAPDH and quantified using NIH ImageJ densitometry software, with the results expressed as a fold change vs. control.

### RNA extraction, reverse transcription, and real-time PCR

Total cellular RNA was extracted from fibroblasts using PureLink RNA Mini Kit (cat # 12183025, Life Technologies, Carlsbad, CA). DNAse 1-treated RNA (1 µg) was reverse–transcribed into cDNA using a high-capacity iScript cDNA synthesis kit (Bio-Rad Laboratories), which provides sensitive two-step RT-qPCR in a 20 µL reaction. The gene expression for IRE1α, XBP-1, ATF4, CHOP, NF-κB, IL-6 & Col-1 were quantified and analyzed using SYBER green qPCR master mix (Bio-Rad Laboratories) on an Applied Biosystems RT-PCR machine (QuantStudio 7 Pro). L27 housekeeping gene was used to normalize all mRNA levels calculated using the manual 2^-(ddCT) method and expressed as a fold change vs. control.

### Hydroxyproline assay

The collagen production in cell culture was measured with an established Hydroxyproline assay protocol as previously described (Woessner, 1961; Rhaleb et al., 2001). Briefly, HCFs were seeded in a 6-well plate, grown in complete basal media containing 10% FBS and growth factor until they reached 100% confluence, and then incubated in serum-free media for 24 h. Fibroblasts were cultured in fresh 0.4% FBS containing 0.15 mmol/L, L-ascorbic acid, 1 μM captopril, and treated with Ac-SDKP (10 nM) and TUDCA (500 μg/mL) for 30 min before the addition of TM for 48 h (Rhaleb et al., 2001). At the end of the experiment, media was collected and allowed to precipitate in two volumes of absolute ethanol at −20°C for at least 24 h. We centrifuged the samples at 14,000–16,000 rpm (18,800–25,700 rcf) for 30 min after precipitation. The pellet was air-dried and resuspended in 500 μL of 6N HCl for 16 h at 110°C in the React-Therm III- Heating Module (Pierce). A savant was used to dry the hydrolyzed samples (SPD131DDA, Thermo-Scientific, Asheville, NC). After dissolving the residue in 1 mL of water, a color-based reaction described by Stegemann and Stalder (Stegemann and Stalder, 1967) using a standard curve for 0–5 mg hydroxyproline determined the overall hydroxyproline content. After scraping the plates containing 100 µL of lysis buffer and preparing the lysate, we measured the protein concentration using the previously described Bradford method (Peng et al., 2014). Assuming that collagen contains 13.5% Hydroxyproline, we expressed the results as micrograms of collagen produced in the medium per milligram of fibroblast protein (Chiariello et al., 1986).

### Inflammatory cytokine IL-6 measured by ELISA

We performed an enzyme-linked immunosorbent assay (ELISA) to measure cytokine IL-6 in cell supernatant collected after 24 h of treatment. Briefly, we seeded the fibroblasts in a 6-well plate and treated them with Ac-SDKP (10 nM) and TUDCA (500 μg/mL) for 30 min before adding TM (0.25 μg/mL). We collected the cell supernatant after 24 h. According to the manufacturer’s protocol, the cytokine IL-6 levels were detected using a commercially available ELISA kit (Thermo Fisher Scientific, Waltham, MA, USA). The cell supernatant was diluted with the sample diluent equally (1:1 dilution), and 100 µL in total volume was used to measure IL-6 concentration according to the manufacturer’s recommendations. We performed all experiments in triplicate, and the concentrations of cytokine IL-6 were expressed as pg/mg protein.

### Statistical analysis

SAS/STAT software, Version 9.4 of the SAS System for Windows, was used for statistical analysis. All the data are expressed as means ± standard errors, with the two-sample Wilcoxon test with a Hochberg’s correction for multiple testing used for group comparison and the Fligner-Polercello correction used for unequal variances. The data were presented as fold change vs. control. An adjusted *p*-value <0.05 was considered significant.

## Results

### Effect of Ac-SDKP on TM-induced cell toxicity and viability

**FIGURE 1:**
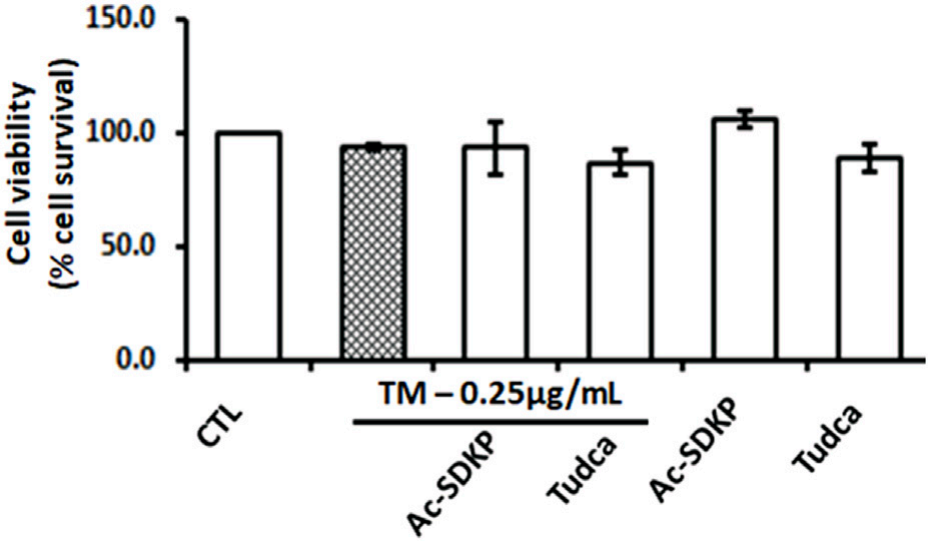
Effects of Ac-SDKP on Tunicamycin (TM)-induced cell toxicity and viability on human cardiac fibroblasts. The minimal toxicity and maximum cell viability were observed with TM at 0.25 μg/mL for 24-hour treatment, which was considered when treating the cells for all corresponding experiments (*n* = 3).

The cell toxicity and viability assays show no significant impact on cell death or loss in cell viability despite robust ER stress induction by TM at a dose of 0.25 μg/mL in the presence or absence of Ac-SDKP (10 nM) and TUDCA (500 μg/mL) in cardiac fibroblasts (Figure 1 and Supplementary Figure S2). The cells in each well were counted, and the representative images were captured under an inverted microscope, as shown in Supplementary Figures S1, S2.

### Effect of Ac-SDKP on TM-induced ER stress UPR signaling

**FIGURE 2:**
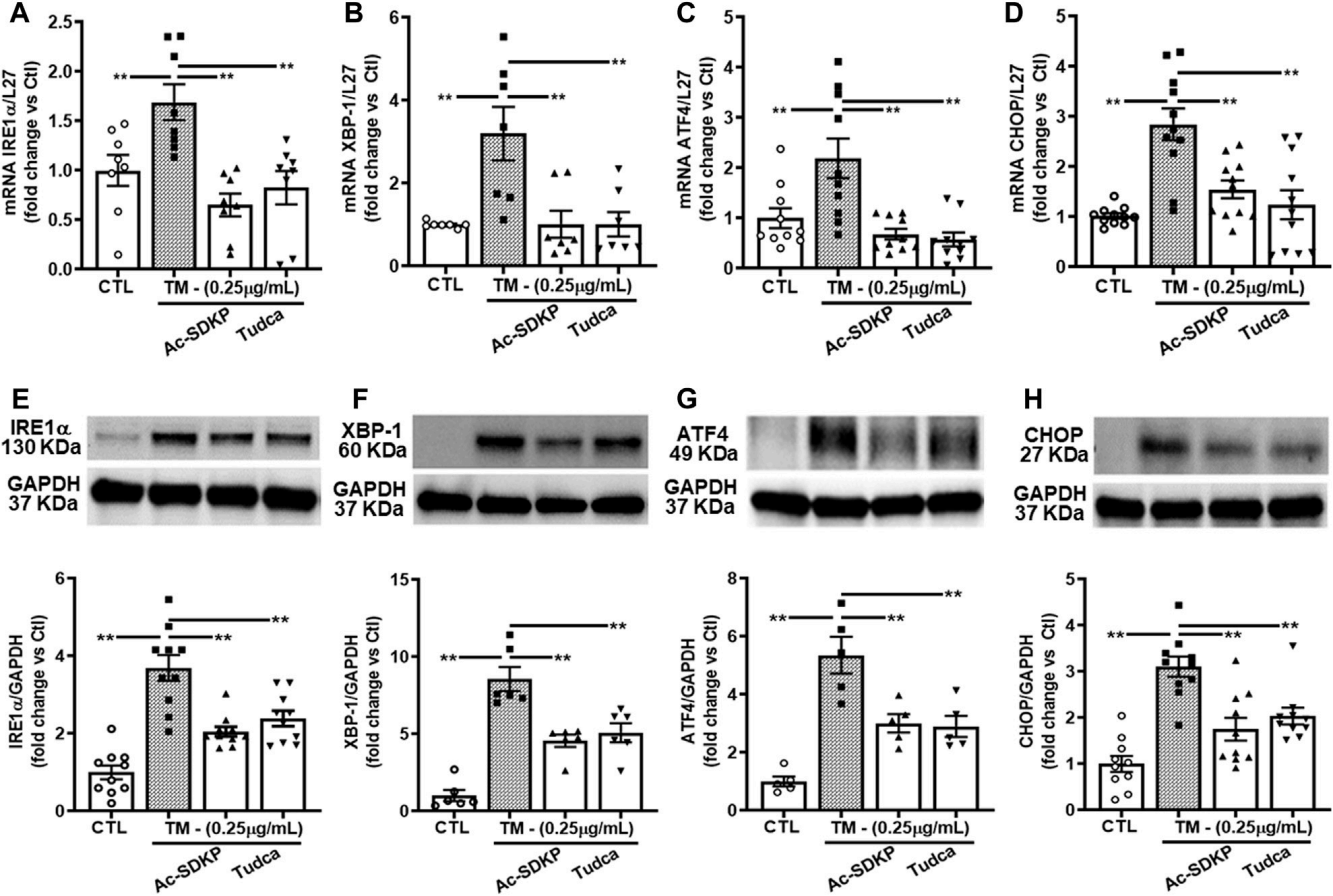
Expression of ER stress markers and UPR activation in human cardiac fibroblasts treated with Tunicamycin (TM) in the presence or absence of Ac-SDKP. The samples were collected and processed at 4 h for RNA extraction and at 24 h for protein analysis. **(A–D)** The mRNA levels for respective genes IRE1α, XBP-1, ATF4, and CHOP normalized with internal control L-27 (*n* = 5–10). **(E–H)** We probed specific antibodies raised against IRE1α, XBP-1, ATF4, and CHOP, as seen on representative Western blots (*n* = 5–10). The density of corresponding band intensities was measured and quantified using ImageJ analysis software and adjusted with GAPDH as a reference standard. Data are expressed as mean ± SEM, and values were expressed as fold change vs. control. **p ≤ 0.001.

We measured the TM-induced ER stress-activated UPR sensor, which upregulates transcription factor proteins and their downstream signaling pathways in HCFs. We observed robust ER stress induction by TM at 0.25 μg/mL in HCFs. ER stress-induced UPR activation upregulates IRE1α, XBP-1, and subsequent ATF4 required for CHOP induction. We analyzed mRNA expression for IRE1α, XBP-1, ATF4, and CHOP genes by RT-PCR and observed that Ac-SDKP and TUDCA attenuated the significantly increased mRNA expression (Figures 2A–D). Ac-SDKP and TUDCA significantly inhibited the increase in TM-induced ER stress markers and its associated UPR chaperon proteins IRE1α, XBP-1, ATF4, and CHOP (Figures 2E–H).

### Ac-SDKP decreases TM-induced NF-κB and Col-1 expression

**FIGURE 3:**
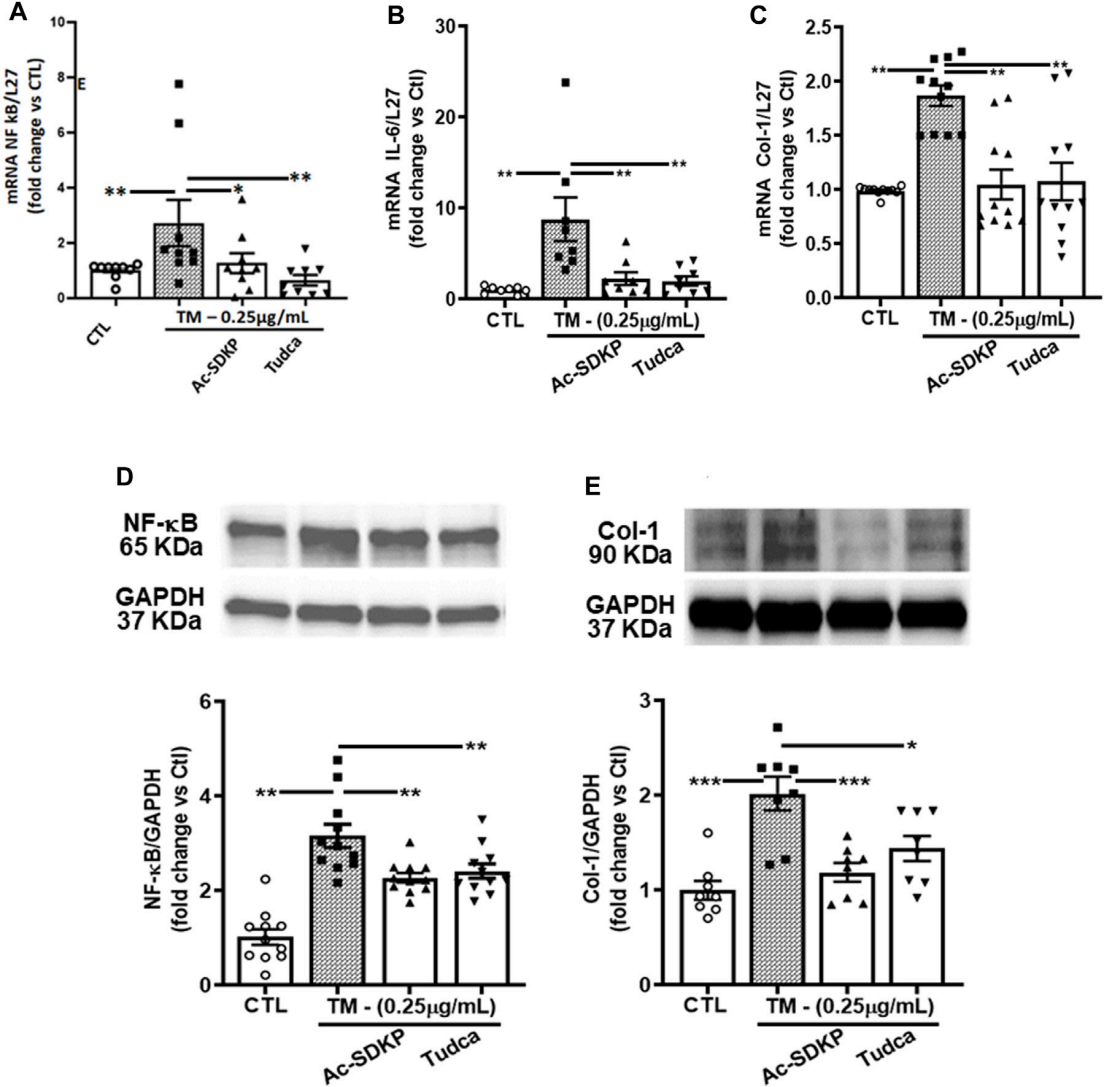
Effects of Ac-SDKP on TM-induced NF-κB, IL-6, and Col-1 expression. **(A–C)** The mRNA level for the corresponding genes (NF-κB, Col-1, and IL-6) was measured by RT-PCR inhibited by Ac-SDKP (*n* = 5–10). **(D)** Ac-SDKP blocked the induction of inflammation, as measured by Western blot, by inhibiting CHOP-mediated NF-κB expression, which was required for TM-induced ER stress (*n* = 5–10). **(E)** Similarly, the elevated Col-1 protein expression, a marker for fibrosis, was alleviated by Ac-SDKP (*n* = 5–10). Data are expressed as mean ± SEM, and values are represented as fold change vs. control. *p ≤ 0.01, **p ≤ 0.001.

Consistent with the above results, the mRNA expressions for NF-κB, IL-6, and Col-1 induced by TM were attenuated significantly by Ac-SDKP and TUDCA (Figures 3A–C). Similarly, at the protein level, Ac-SDKP inhibited the TM-induced NF-κB expression (Figure 3D). Lastly, we tested the underlying marker for collagen production, such as Col-1 expression in cardiac fibroblasts. Parallel to NF-κB, pretreatment with Ac-SDKP significantly reduced the TM-induced increase of Col-1 expression (Figure 3E). Additionally, we employed the scrambled peptide ‘PKDS’ to validate the specificity of Ac-SDKP inhibitory effect in rat cardiac fibroblasts prepared following our established protocol (Rhaleb et al., 2001; Zhuo et al., 2007; Peng et al., 2012). The scrambled peptide failed to inhibit endothelin-1-induced collagen production compared to Ac-SDKP (Supplementary Figure S3).

### Effects of Ac-SDKP on TM-induced NF-κB activation and Col-1 expression in cardiac fibroblasts with CHOP knockdown

**FIGURE 4:**
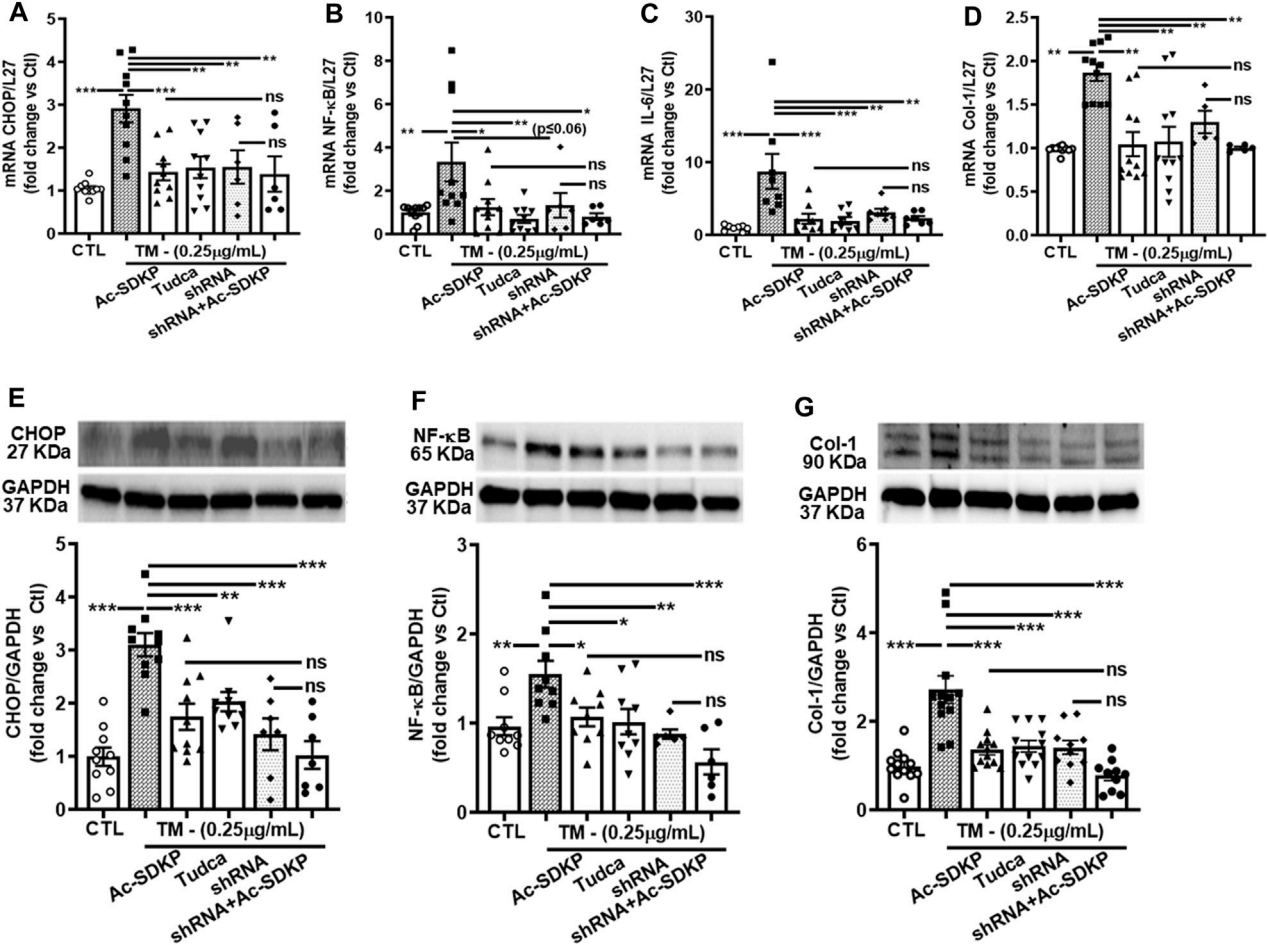
Effects of Ac-SDKP on TM-induced NF-κB activation, IL-6, and Col-1 expression in cardiac fibroblasts with CHOP-knockdown. Fibroblasts were knocked down with CHOP-specific shRNA for the corresponding time per the manufacturer’s protocol, and samples were processed for mRNA and protein expression (**A, E**). mRNA expression measured by RT-PCR and normalized with L-27 as an internal control followed by protein analysis by Western blot quantified and normalized with GAPDH as an internal control **(B–D, F, G)**. shRNA CHOP-knockdown results in additive effects of NF-κB and Col-1 expression by Ac-SDKP at both protein and RNA levels (*n* = 5–10), trending towards significance statistically. Data are expressed as mean ± SEM, and values are represented as fold change vs. control. *p ≤ 0.01, **p ≤ 0.001, ***p ≤ 0.0001.

**FIGURE 5:**
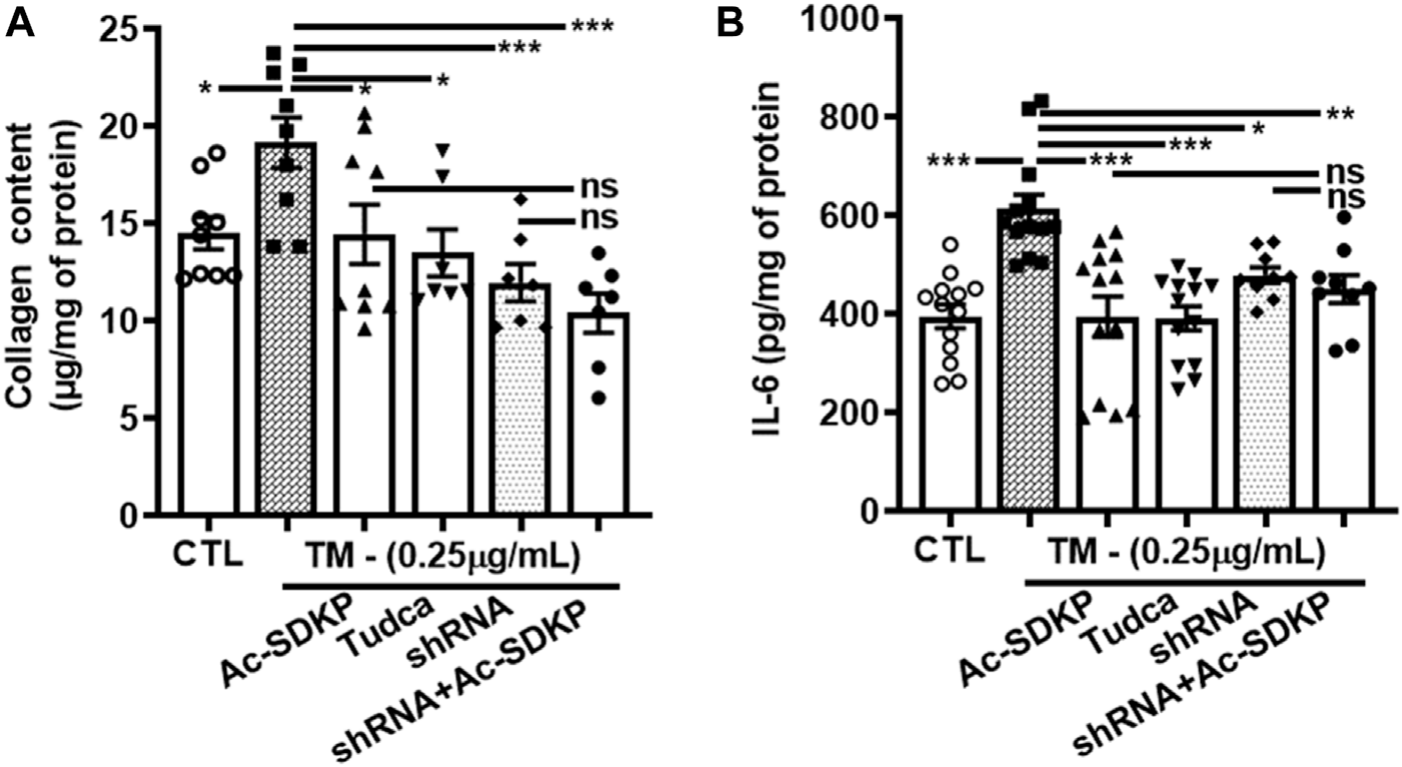
CHOP-knockdown maintained the inhibitory effects of Ac-SDKP on collagen content and cytokine IL-6 levels in TM-stimulated cardiac fibroblasts. Collagen contents and inflammatory cytokine IL-6 measured in cell supernatant media collected at 24 and 48 h, respectively, from the shRNA CHOP-knockdown fibroblast treated with TM. The profound increase in IL-6 **(A)** and collagen content **(B)** represents a maintained an inhibitory effects of Ac-SDKP in CHOP-knockdown fibroblast stimulated with TM (*n* = 6). Data are expressed as mean ± SEM, *p ≤ 0.01, **p ≤ 0.001, ***p ≤ 0.0001.

We used specific shRNA lentiviral plasmids to knock down CHOP in cardiac fibroblast (Figures 4A, E). Interestingly, the inhibitory effects of Ac-SDKP on NF-κB and Col-1 expression were sustained at both mRNA (Figures 4B–D) and protein levels (Figures 4F, G) in CHOP-specific knockdown cells treated with TM. In addition to Col-1, we assessed the inhibitory effects of Ac-SDKP by examining the expression of another fibrotic marker, such as αSMA, following TM treatment. Illustrated in Supplementary Figures S4A, B, a reduction of the elevated expression of αSMA is most likely the inhibitory effect of Ac-SDKP in CHOP-specific knockdown cells treated with TM. As shown in our data, the attenuation in NF-κB expression and profibrotic αSMA expression by Ac-SDKP are most likely mediated by CHOP inhibition. Additionally, we observed that Ac-SDKP inhibitory effects was maintained consistently in collagen contents and inflammatory cytokine IL-6 levels in cell supernatant media collected after treatments in CHOP-specific knockdown cardiac fibroblast treated with TM (Figures 5A, B).

## Discussion

Cardiac fibrosis develops as a result of heightened levels of extracellular matrix components, notably collagen fibers, synthesized by cardiac cells. Collagen deposition occurs when the heart is exposed to various injuries, such as myocardial infarction, myocarditis, pressure overload, and/or intrinsic or extrinsic stresses (Peng et al., 2001). The accumulation of unfolded proteins in the ER lumen results in ER stress and has been implicated in the pathogenesis of many diseases, including hypertension and cardiac fibrosis. Fibroblasts constitute the major part of the cells in the heart (Kurose, 2021). The findings from the current study demonstrate the induction of ER stress in HCFs in response to TM stimulation, represented by a substantially enhanced expression of ER stress UPRs sensor IRE1α, chaperon’s molecules such as XBP-1, ATF4, and CHOP at both transcription and translational level (Figures 2A–H). The effects of TM were void of any apparent effect on cell survival rate. Treating the fibroblasts with Ac-SDKP modulates ER stress induced by TM and inhibits the overexpressed IRE1α, XBP-1, ATF4, and CHOP at both protein and mRNA levels. In response to TM stimulation, the increased expression of chaperone molecules is due to the accumulation of unfolded proteins in the ER lumen. Therefore, cells trigger UPR sensor molecules to restore normal ER function. Our results show that the overexpressed CHOP-dependent activation of inflammatory transcription factor NF-κB increased significantly in response to TM stimulation. NF-κB, an essential mediator of the inflammatory response, is activated when cells cannot alleviate unfolded proteins to restore homeostasis in the ER lumen. In the present study, we showed that Ac-SDKP, TUDCA, or CHOP-shRNA inhibited 24 h.-induced NFκB expression, thereby decreasing its availability to phosphorylating kinases and translocation to the nucleus; others have shown similar regulation of NFκB expression under different experimental conditions (Gunda et al., 2021; Zhou et al., 2021). NF-κB activation is typically rapid and transient, with peak response occurring minutes after stimulation (Zhong et al., 2002; Jiang et al., 2015; Chen et al., 2020). Nevertheless, our findings showing downregulation of NF-κB, IL-6, and collagen production by Ac-SDKP agree with our previous findings in endothelial cells and rat cardiac fibroblasts (Rhaleb et al., 2001; Wang et al., 2010; Zhu et al., 2016; Zhang Y. et al., 2017). Ac-SDKP inhibits the overexpressed CHOP-dependent NF-κB signaling pathway induced by TM at both mRNA and protein levels (Figures 3A, D). Attenuating NF-κB expression by Ac-SDKP results in the decreased secretion of inflammatory cytokine IL-6 in cell culture media (Figure 5B). Per our hypothesis, we also expected to see increased collagen production in response to prolonged ER stress and inflammation. Our previous studies showed the dose-dependent inhibitory effects of Ac-SDKP in cardiac fibroblast proliferation and collagen synthesis (Peng et al., 2001; Peng et al., 2010). Our data showed that Ac-SDKP inhibited TM-stimulated collagen production by cardiac fibroblasts at both mRNA and protein levels (Figures 3C, E). CHOP is a molecular chaperon strongly expressed in response to stress; however, under normal physiological conditions, CHOP is expressed ubiquitously at shallow levels in most cells (Lei et al., 2017). Therefore, to explore and investigate CHOP’s influence on inflammation and collagen production in cardiac fibroblasts, we used CHOP-specific lentiviral plasmids to knock down the CHOP expression. Data presented here show that specific knockdown of CHOP results in an inhibition of NF-κB and Col-1 expression (Figures 4A–G) and decreased inflammatory cytokine IL-6 and collagen production in cell media (Figures 5A, B). However, we did not observe any additional significant changes at transcription levels in CHOP-specific mRNA knockdown cells. Consistent with Col-1 data, Ac-SDKP treatment reduces collagen production by HCFs. Fibroblasts are abundantly found in the connective tissues of most organs, including the heart. Cardiac fibroblasts have diverse functions and are activated in response to cardiac injury, myocardial infarction, myocarditis, and pressure overload (Brilla and Maisch, 1994; Rhaleb et al., 2001). The phenotypic and physiological changes in cardiac fibroblasts are the primary cause of cardiac remodeling, leading to heart failure. Cardiac fibroblasts are the prime source of cardiac inflammation and fibrosis in response to stress, myocardial injury, aberrant ER homeostasis, calcium imbalance, and ATP reduction (Doroudgar et al., 2009; Jin et al., 2017; Blackwood et al., 2019; Stauffer et al., 2020). Many studies have shown that UPR activation and its chaperone proteins are crucial in cardiac inflammation and fibrosis. They are the prime causative factors in hypertension and cardiac remodeling leading to heart failure (Brilla and Maisch, 1994; Liu et al., 1997; Rhaleb et al., 2001; Hotamisligil, 2010; Thorp, 2012; Liu and Dudley, 2014; Zhang C. et al., 2017; Szymanski et al., 2017; Amen et al., 2019). Our finding indicates that Ac-SDKP restores the normal ER function of cardiac fibroblasts by inhibiting the UPRs sensor chaperon proteins, thus protecting the fibroblasts from TM-induced inflammation and collagen overexpression. We believe this is the first study showing that Ac-SDKP attenuates UPR sensor IRE1α, chaperon proteins (XBP-1, ATF4 & CHOP), CHOP-dependent NF-κB signaling and Col-1 expression in cardiac fibroblasts. Three main UPR sensor pathways exist, namely PERK, IRE1α, and ATF6 and each sensor plays a key role independently or in conjunction to resolve and restore normal ER function. When the cells are under prolonged ER stress, the cells activate multifactorial mechanisms such as inhibition of protein translation, induction of ER chaperon gene transcription, and activation of the ER-associated proteasome degradation (ERAD) pathway to restore normal cell function in the ER lumen (Ayaub et al., 2016). Under ERAD, the cells activate CHOP-dependent on an essential inflammatory transcriptional NF-κB signaling, which regulates many genes involved in inflammation, fibrosis, apoptosis, and cell death (Pahl and Baeuerle, 1995; Jiang et al., 2003; Deng et al., 2004; Tam et al., 2012; Willy et al., 2015).

The data presented in this study showed that Ac-SDKP inhibited TM-induced upregulated UPR sensors, chaperon proteins, and CHOP-mediated inflammatory NF-κB signaling and extracellular matrix protein Col-1 expression in HCFs. We and others have previously shown that Ac-SDKP binds specifically to cardiac cells, including fibroblasts and other cell types (Cheviron et al., 2000; Zhuo et al., 2007; Sonkawade et al., 2023); however, complete characterization and cloning of the putative receptor(s) for Ac-SDKP still require more molecular and biochemical studies toward substantially improving our understanding of the mechanism(s) of action of Ac-SDKP. Our findings indicate that UPR transcriptional factor CHOP plays a pivotal role in ER stress-induced cardiac inflammation and fibrosis. CHOP is pro-apoptotic, whereas NF-κB is an anti-apoptotic transcription factor, and these paradoxical observations reported may be due to specific cell type response to ER stress (Nozaki et al., 2001). According to the current results, IRE1α, a UPR sensor, requires CHOP for NF-κB activation in response to TM stimulation in HCFs. Other studies have reported that CHOP-mediated NF-κB activation involves UPR sensor PERK and/or IRE1α in mouse embryonic fibroblasts (Tam et al., 2012). However, our findings showed no significant difference in PERK expression between the groups in response to TM stimulation. Indeed, in conjunction with IRE1α, UPR sensor PERK and ATF6 signals in chaperone loops trigger other CHOP-independent NF-κB signaling pathways, which need further investigation.

**FIGURE 6:**
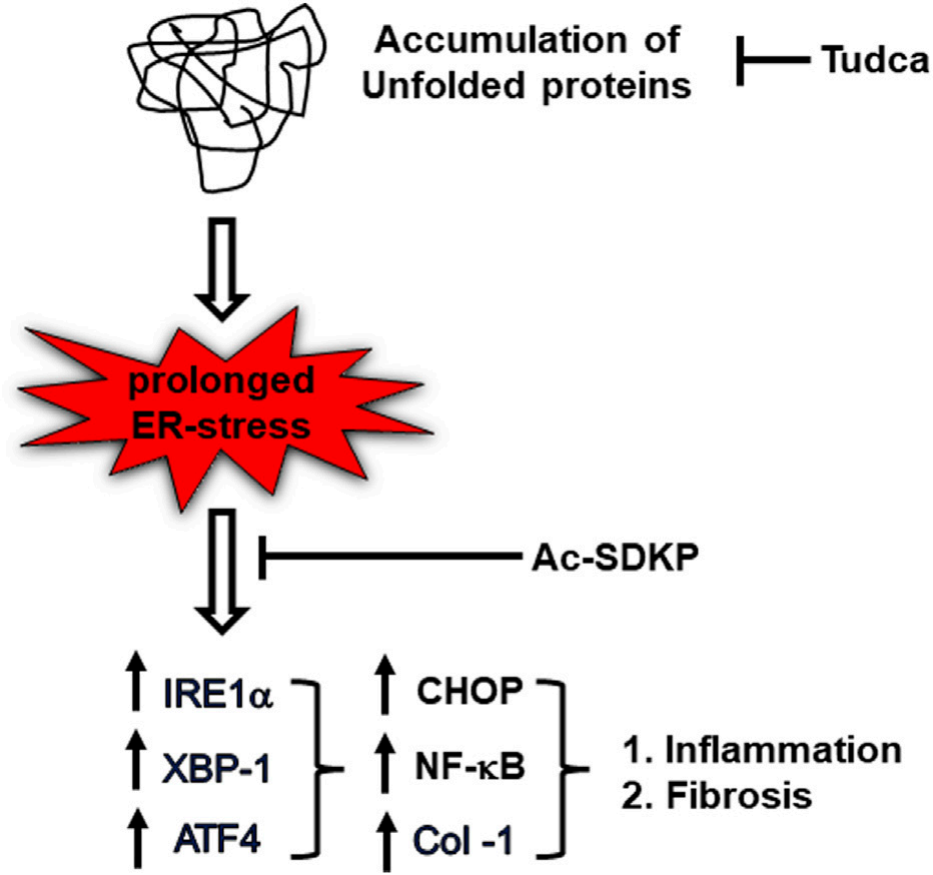
Schematic diagram representing ER stress-induced UPR activation, NF-κB signaling upregulation, and collagen production in TM-stimulated cardiac fibroblasts. Ac-SDKP downregulates ER stress-induced inflammation and collagen content in TM-stimulated cardiac fibroblast. Tudca is shown as a representative ER-stress inhibitor.

In summary, we suggest possible mechanisms that link UPR sensor IRE1α, required for CHOP-dependent NF-κB activation and Col-1 expression progressing to inflammation and fibrosis; Ac-SDKP and ER stress blockers inhibit these factors in HCFs (Figure 6). The cardiac fibroblasts might ramp up collagen production as a protective mechanism or as part of a reparative process to potentially address any damage or stress-induced changes within the cardiac tissue. Nevertheless, our study further supports Ac-SDKP as a potential therapeutic agent in cardiovascular disease intervention and management. The anti-fibrotic effect of Ac-SDKP could be beneficial in treating patients with cardiac remodeling and cardiac dysfunction; further studies are required to establish its role as an anti-inflammatory and anti-fibrotic molecule in translational or clinical setups. Eventually, investigation of other UPR signaling pathways involved in inflammation and fibrosis will help diagnose and treat limited cardiovascular disease and open new therapeutic strategies in other inflammatory and metabolic disorders.

### Limitations of the study

Our study demonstrates that Ac-SDKP regulates UPR sensor IRE1α and its downstream chaperone molecules XBP-1, ATF4, and CHOP required for NF-κB activation in response to TM stimulation. However, we cannot exclude the possibility that other UPR signaling pathways also contribute to this inhibitory effect of Ac-SDKP, which needs further investigation.

#### Highlights of the study

Ac-SDKP inhibits 1) increased UPR sensor IRE1α activation, 2) enhanced XBP-1, ATF4, and CHOP transcription factor expression, 3) increased NF-κB signaling, and 4) Col-1 upregulation in cardiac fibroblasts stimulated with TM. Ultimately, Ac-SDKP attenuates increased inflammatory cytokine IL-6 and collagen production.

## Data Availability

The original contributions presented in the study are included in the article/Supplementary Material, further inquiries can be directed to the corresponding author.

## Abbreviations

ER, endoplasmic reticulum; UPRs, unfolded protein responses; Ac-SDKP, N-Acetyl-Ser-Asp-Lys-Pro; TUDCA, tauroursodeoxycholic acid; HCFs, human cardiac fibroblasts; ACE, angiotensin converting enzyme; ACEi, ACE inhibitor; CHOP, C/EBP homologous protein; FBS, fetal bovine serum; IER1, inositol-requiring enzyme type 1; PERK, protein kinase RNA-like endoplasmic reticulum kinase; ATF6, activating transcription factor 6; Col-1, type-1 collagen; NF-κB, nuclear factor kappa B, IL-6, interlukin-6; TNF-α, Tumor necrosis factor alpha.
