# Supplementary figures and images for: Ac-SDKP attenuates ER stress-stimulated collagen production in cardiac fibroblasts by inhibiting CHOP-mediated NF-κB expression
Source: Front Pharmacol. 2024 Mar 1;15:1352222. doi: 10.3389/fphar.2024.1352222 (PMC10940518; doi:10.3389/fphar.2024.1352222)

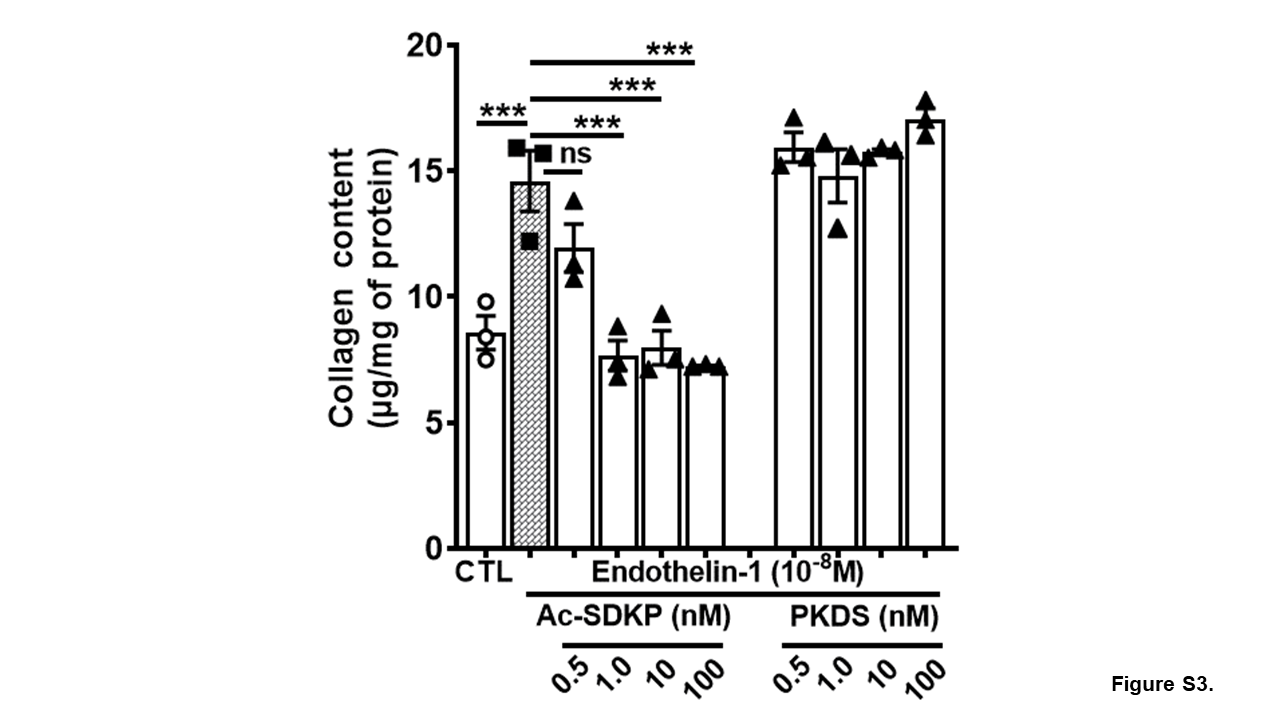

Supplement: Supplementary file 1 [file Image6.TIF]

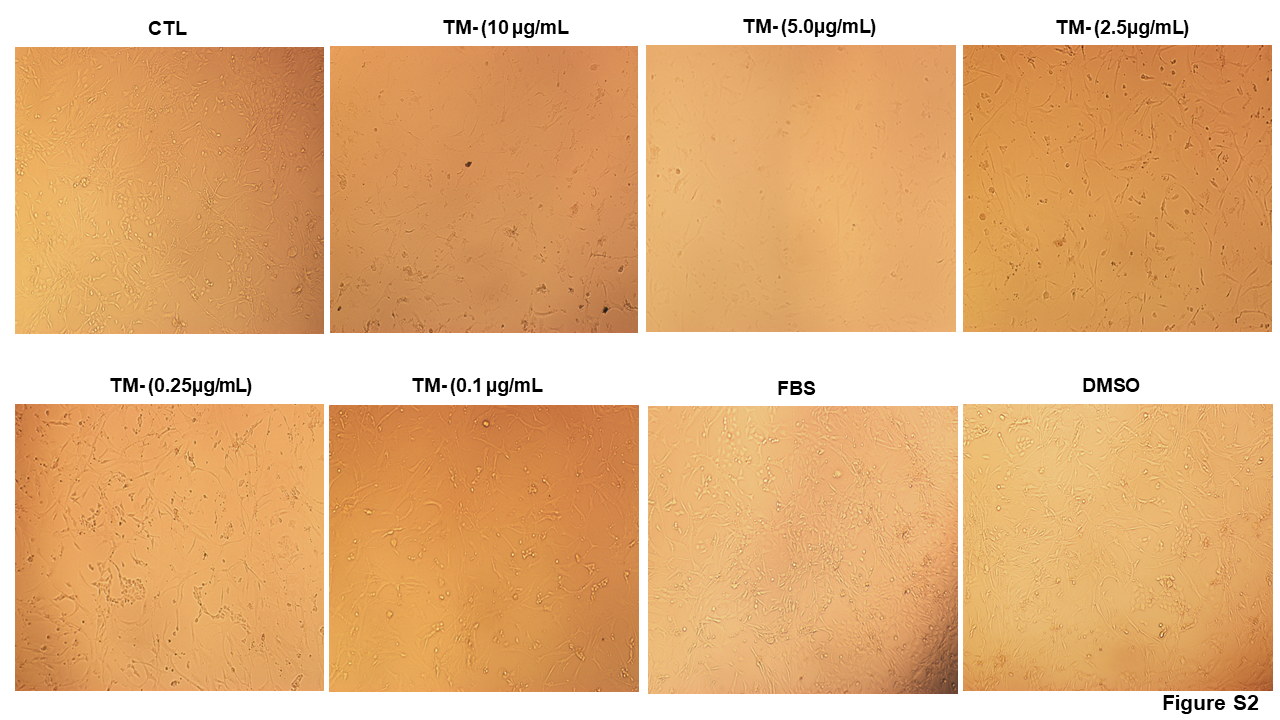

Supplement: Supplementary file 2 [file Image3.tif]

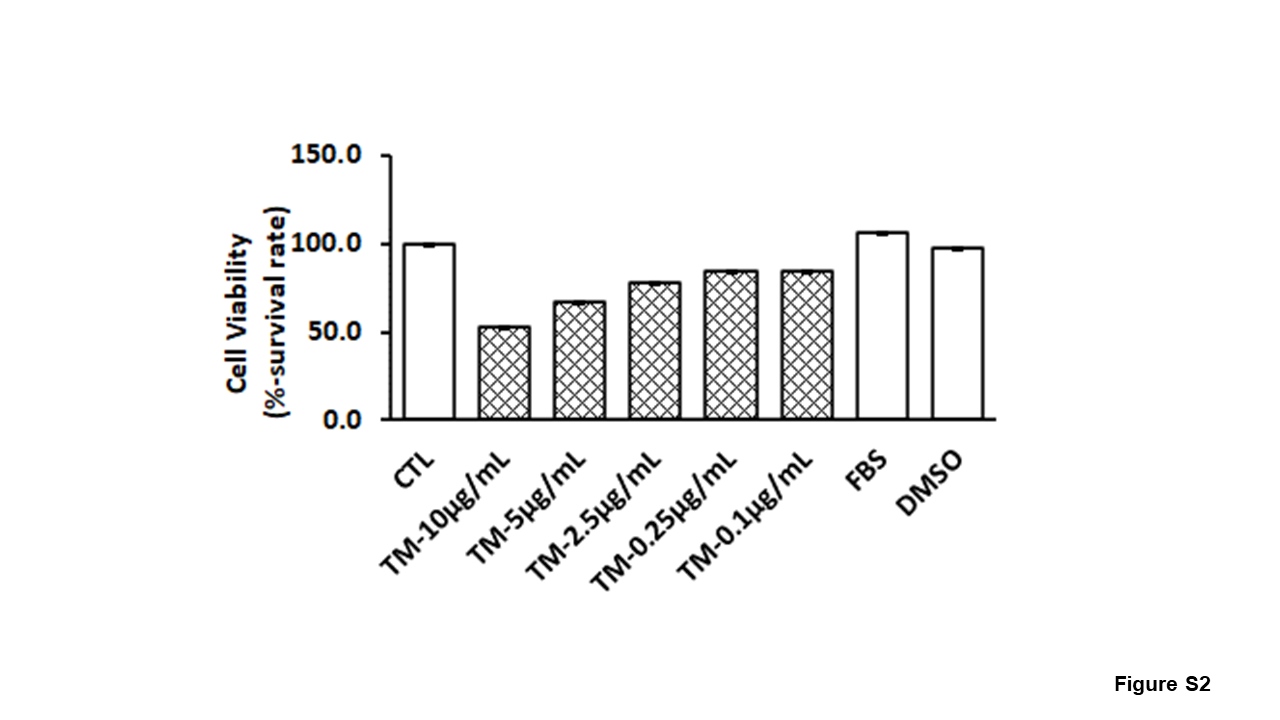

Supplement: Supplementary file 3 [file Image4.tif]

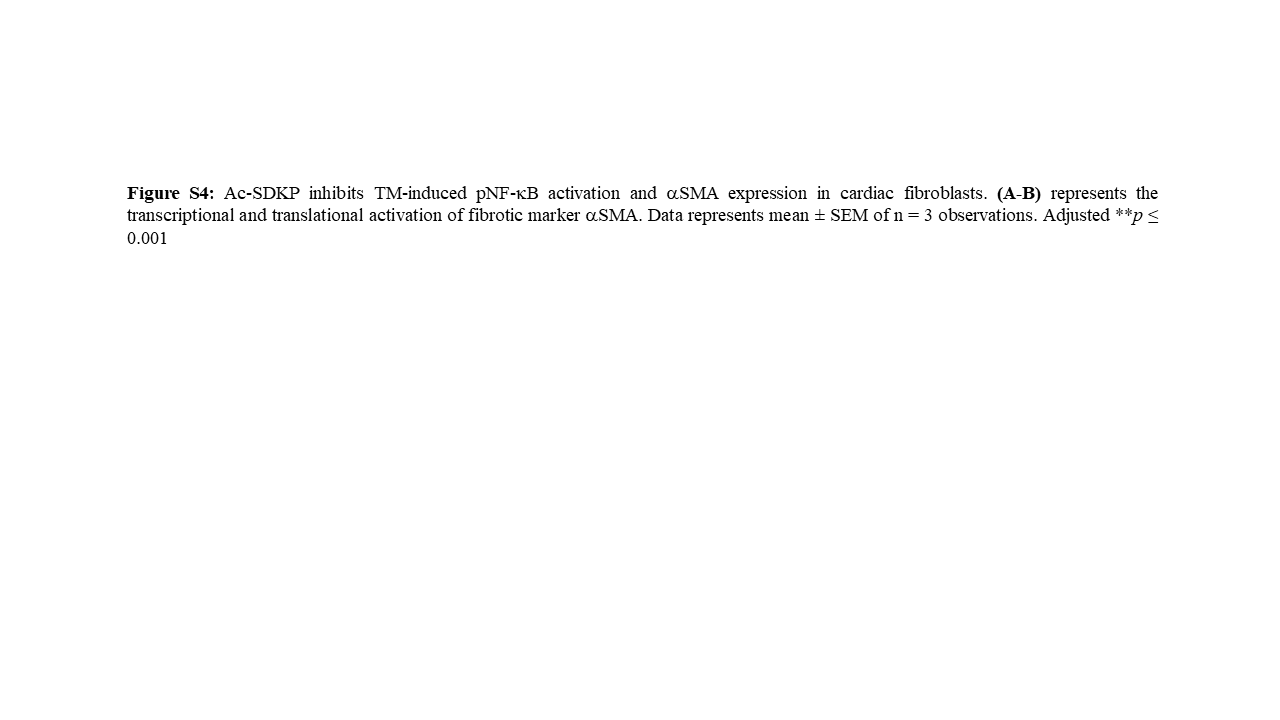

Supplement: Supplementary file 4 [file Image9.TIF]

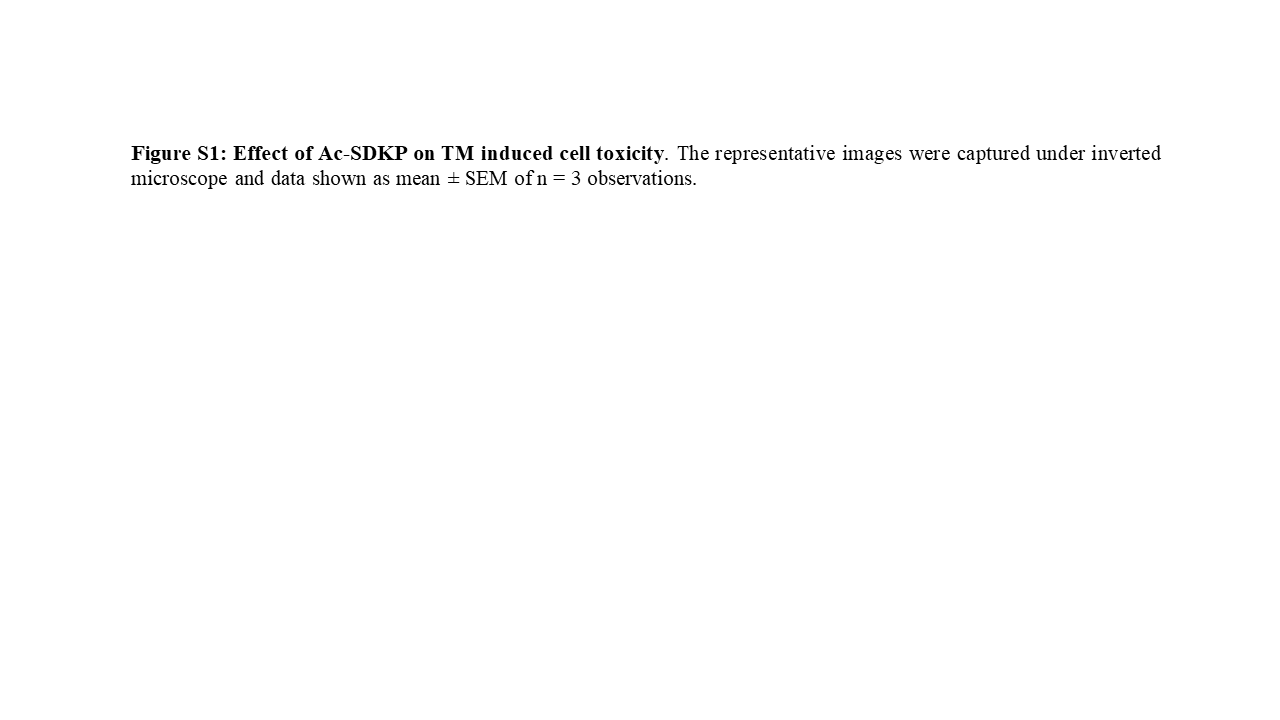

Supplement: Supplementary file 5 [file Image2.TIF]

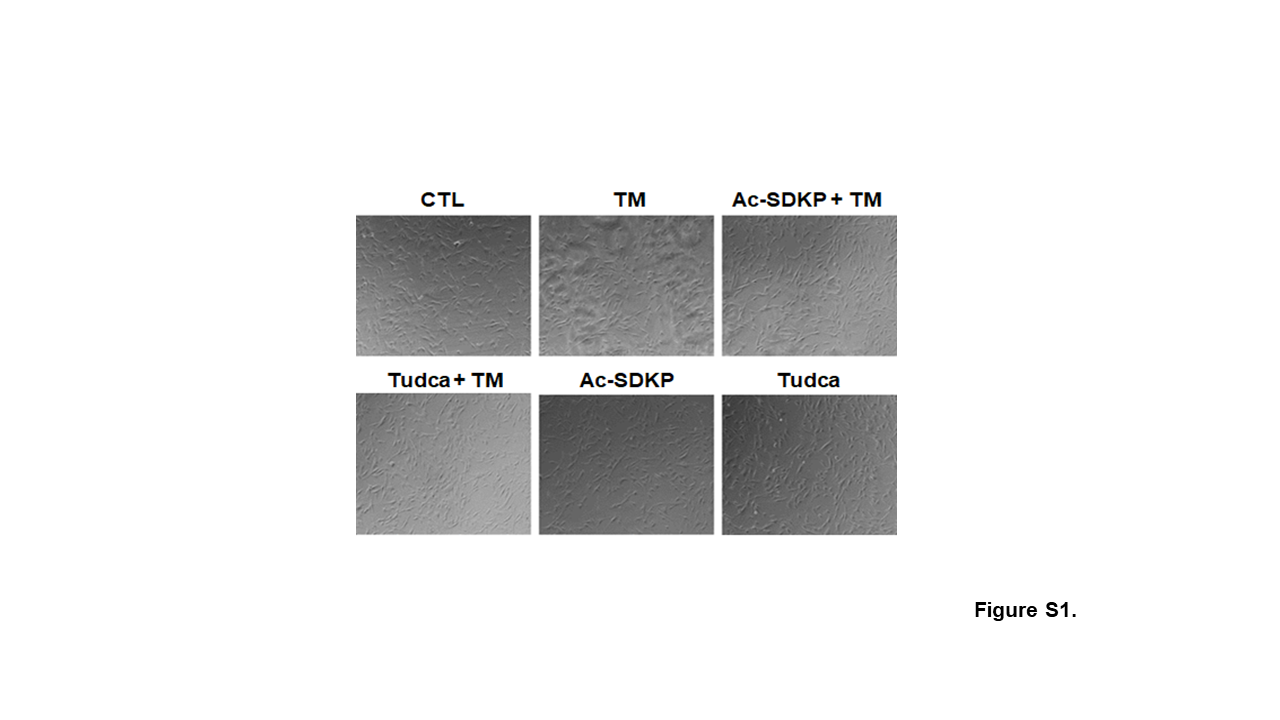

Supplement: Supplementary file 6 [file Image1.TIF]

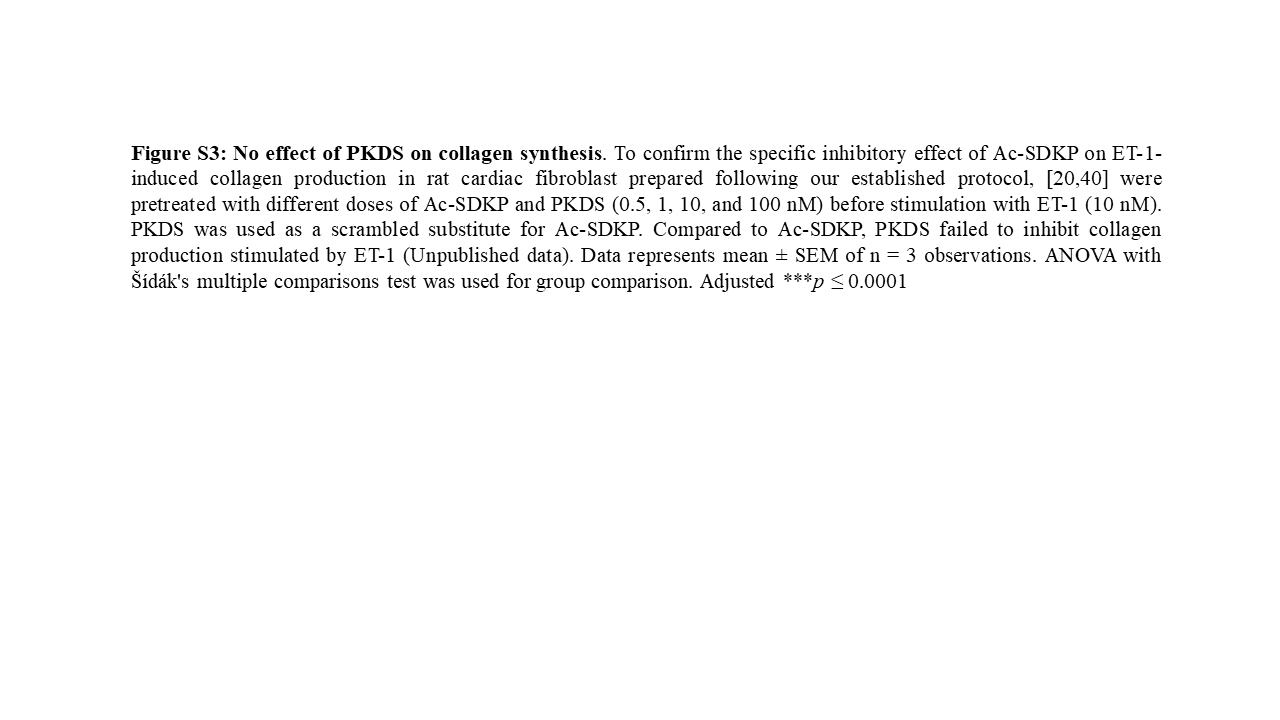

Supplement: Supplementary file 7 [file Image7.TIF]

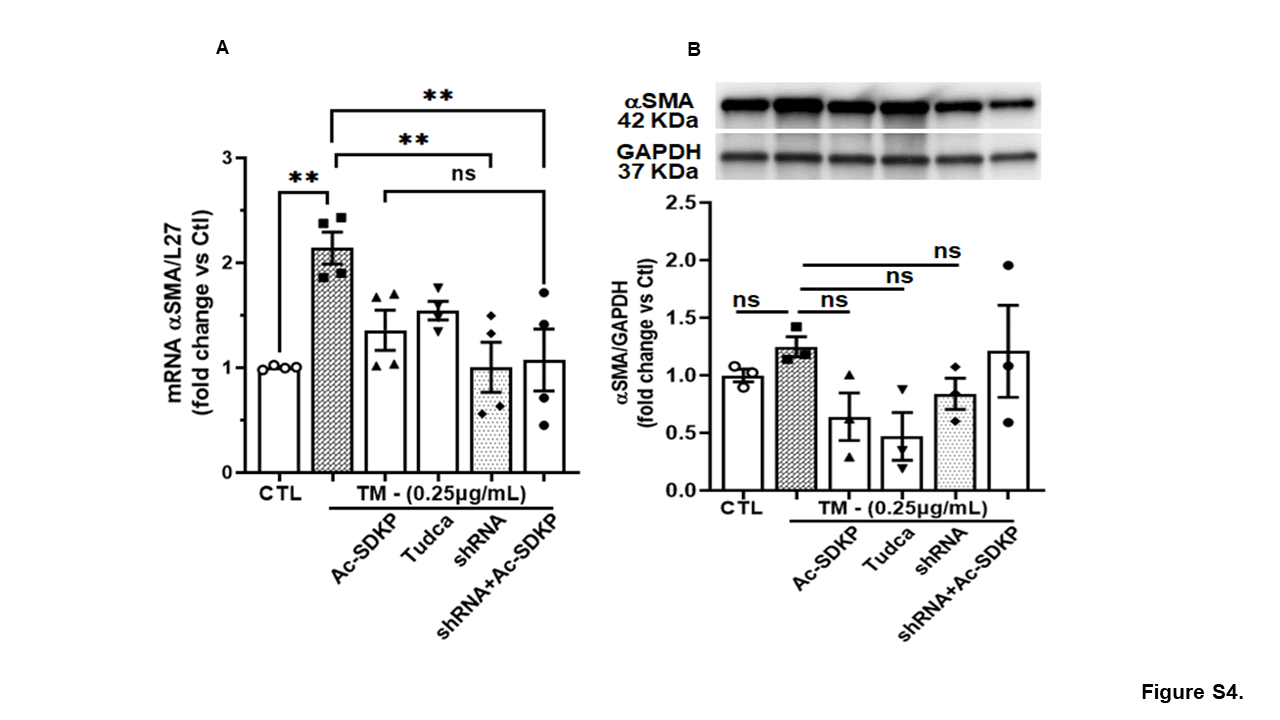

Supplement: Supplementary file 8 [file Image8.TIF]

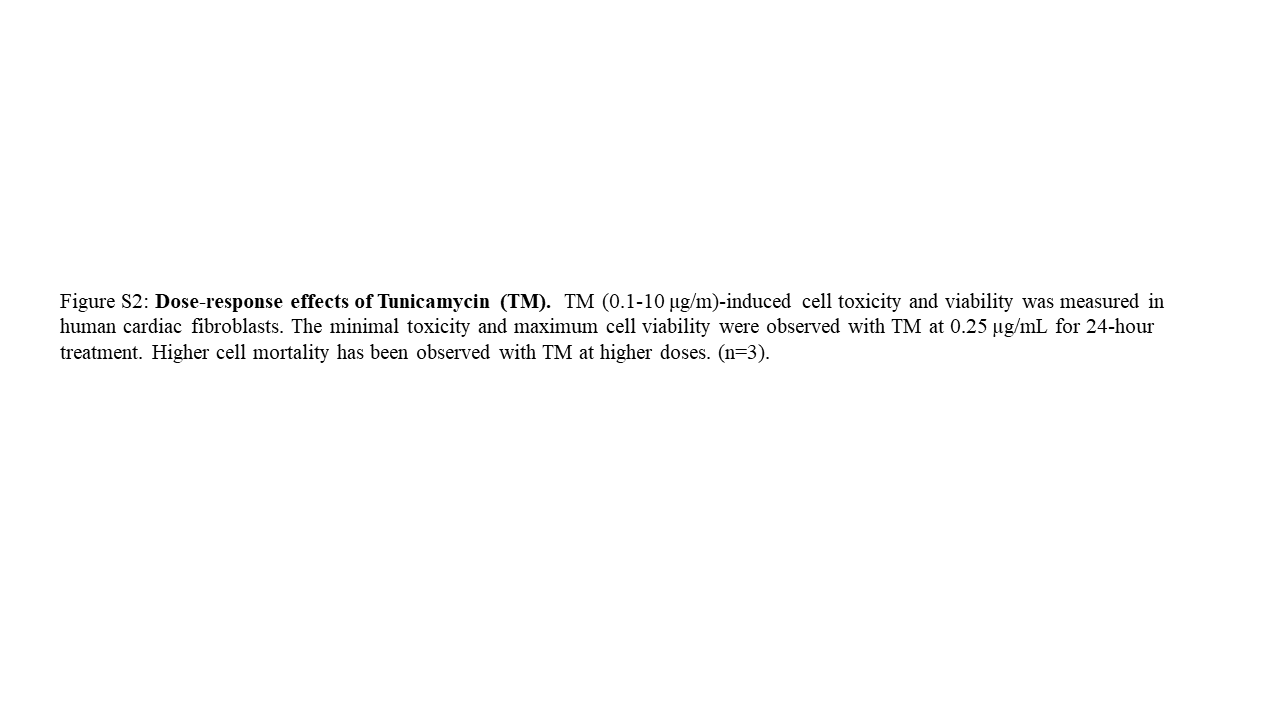

Supplement: Supplementary file 9 [file Image5.TIF]
